# Hand therapy interventions for the prevention of chemotherapy-induced peripheral neuropathy of the hands in patients with pancreatic cancer

**DOI:** 10.1093/oncolo/oyae285

**Published:** 2024-10-22

**Authors:** Gayle S Jameson, Cynthia Cooper, Courtney Snyder, Sharon M Buchanan, Terri Strelish, Tania Shearon, Daniel D Von Hoff, Curt Bay, Lynne Hull, Lorilyn A Kaatz, Erkut H Borazanci

**Affiliations:** HonorHealth Research Institute, Scottsdale, AZ, United States; Cooper Hand Therapy, Scottsdale, AZ, United States; HonorHealth Research Institute, Scottsdale, AZ, United States; HonorHealth Research Institute, Scottsdale, AZ, United States; HonorHealth Research Institute, Scottsdale, AZ, United States; HonorHealth Research Institute, Scottsdale, AZ, United States; HonorHealth Research Institute, Scottsdale, AZ, United States; A. T. Still University, Mesa, AZ, United States; HonorHealth Research Institute, Scottsdale, AZ, United States; HonorHealth Research Institute, Scottsdale, AZ, United States; HonorHealth Research Institute, Scottsdale, AZ, United States

**Keywords:** chemotherapy-induced peripheral neuropathy, gemcitabine + albumin-bound paclitaxel, gemcitabine + albumin-bound paclitaxel + cisplatin, pancreatic neoplasms, physical and rehabilitation medicine

## Abstract

**Background:**

Chemotherapy-induced peripheral neuropathy (CIPN), a common problem, can impair function and quality of life in patients, potentially limiting chemotherapy and adversely affecting outcomes.

**Methods:**

This trial compared investigational hand therapy intervention (Investigational) compared with a traditional occupational therapy approach (Traditional) to prevent CIPN in patients with pancreatic cancer receiving gemcitabine and albumin-bound paclitaxel containing regimens.

**Results:**

forty-nine patients were enrolled with 40 evaluable for statistical analysis (21 Investigational/19 Traditional). CIPN in the hands was reported in 6 patients (28.6%) in Investigational, and 4 (21.1%) in Traditional *P* = .721. Kaplan-Meier analysis showed a mean time-to-event of 76.0 days (90% CI: 68.5, 83.6), and 75.8 (90% CI: 68.5, 83.2) days respectively, *P* = .614. Fifteen patients in each group (78.9% Traditional, 71.4% in Investigational) were censored as they did not develop CIPN. No correlation was found between CIPN risk and age, sex, BMI, disease stage, performance status, or chemotherapy dose.

**Conclusion:**

Seventy-four percent of patients receiving gemcitabine, albumin-bound paclitaxel, and cisplatin did not develop CIPN of the hands by day 84. There was no statistical difference in time to onset of CIPN between the two groups. Early adaption of occupational therapy may prevent early onset CIPN in chemotherapy patients.

**ClinicalTrials.gov Identifier:**

NCT05374876.

## Teaser

This trial compared investigational hand therapy intervention with a traditional occupational therapy approach to prevent chemotherapy-induced peripheral neuropathy in patients with pancreatic cancer receiving gemcitabine and albumin-bound paclitaxel containing regimens.

**Lessons learned**

- Seventy-five percent of patients receiving gemcitabine plus albumin-bound paclitaxel containing regimen did not develop chemotherapy-induced peripheral neuropathy (CIPN) by day 84 (week 12), suggesting that the development of CIPN may not be of clinical consequence in the majority of patients in the neo-adjuvant setting where shorter courses of chemotherapy are often utilized.
- No difference was seen between the intervention groups, but adaption of occupational therapy may prevent the development of early onset CIPN, and additional study is recommended.

## Discussion

Chemotherapy-induced peripheral neuropathy (CIPN) is caused by the toxicity of multiple anticancer agents including albumin-bound paclitaxel and cisplatin, both commonly used in treating patients with pancreatic cancer, that can result in temporary or permanent damage to the peripheral nervous system or autonomic nervous system. CIPN is a common and debilitating complication associated with neurotoxic chemotherapy that warrants attention; despite many prevention studies, there continues to be a lack of effective preventative treatment options. Rehabilitation research lacks sufficient studies on upper extremity occupational therapy for identifying, preventing, or treating CIPN of the hands. Current evidence suggests that except for duloxetine in the treatment of painful CIPN, pharmacologic agents, herbal remedies, supplemental products, and other modalities lack strong evidence for treating or preventing CIPN.

This pilot, double-blind, randomized clinical trial explored the possible benefits of an investigational hand therapy intervention (Investigational Group) compared with a traditional occupational therapy approach (Traditional Group) to prevent CIPN in pancreatic cancer patients receiving gemcitabine (G) plus albumin-bound paclitaxel (A) containing regimens during the initial 84 days of treatment.

There was no statistical difference in time to onset between the two intervention groups in this prevention study. Notably, 75% of patients receiving gemcitabine plus albumin-bound paclitaxel did not develop CIPN by day 84, an unexpected finding. In addition, all patients except one received a second neurotoxic agent, cisplatin with GA, and 74% (29/39) did not develop CIPN. These findings suggest that CIPN may not be of clinical consequence for most patients in the neo-adjuvant setting where shorter chemotherapy courses are often used.

Both interventions were safe and well-tolerated by patients. The absence of CIPN symptoms in the majority of patients by day 84 underscores the impact of early prevention strategies. With the established benefits of exercise in cancer care, oncology rehabilitation programs are increasingly integrated into standard practice. Future research could expand on this study with a randomized control trial including a non-intervention control group, and incorporating oncology rehabilitation in both intervention groups. Insights from this study may guide future research using neurotoxic chemotherapy agents

## Trial Information

**Table:** 

Disease	Pancreatic cancer
Stage of disease/treatment	Patients with adenocarcinoma of the pancreas who will receive chemotherapy with albumin-bound paclitaxel plus gemcitabine containing combination. The treatment in this study is investigational hand therapy intervention (Investigational Group) compared with a traditional occupational therapy approach (Traditional Group). Refer to Figures 1, 2, 3
Prior therapy	Not specified
Type of study	Pilot, double-blind, randomized clinical trial
Primary endpoint	To determine if hand therapy intervention targeting the nervous system can prevent or delay the time to onset of any grade of CIPN of the hands as measured by NCI CTCAE 4.0 and Patient Reported Outcomes as compared to traditional occupational therapy intervention
Secondary endpoints	To determine whether provocative testing of the upper extremity identifies/predicts patients at risk for CIPN To identify whether scores on the TEN TEST and the QuickDASH correlate to the Common Terminology Criteria for Adverse Events, version 4 (CTCAE-4.0) grades of peripheral neuropathy of the hands in patients who develop CIPN

**Figure 1.**
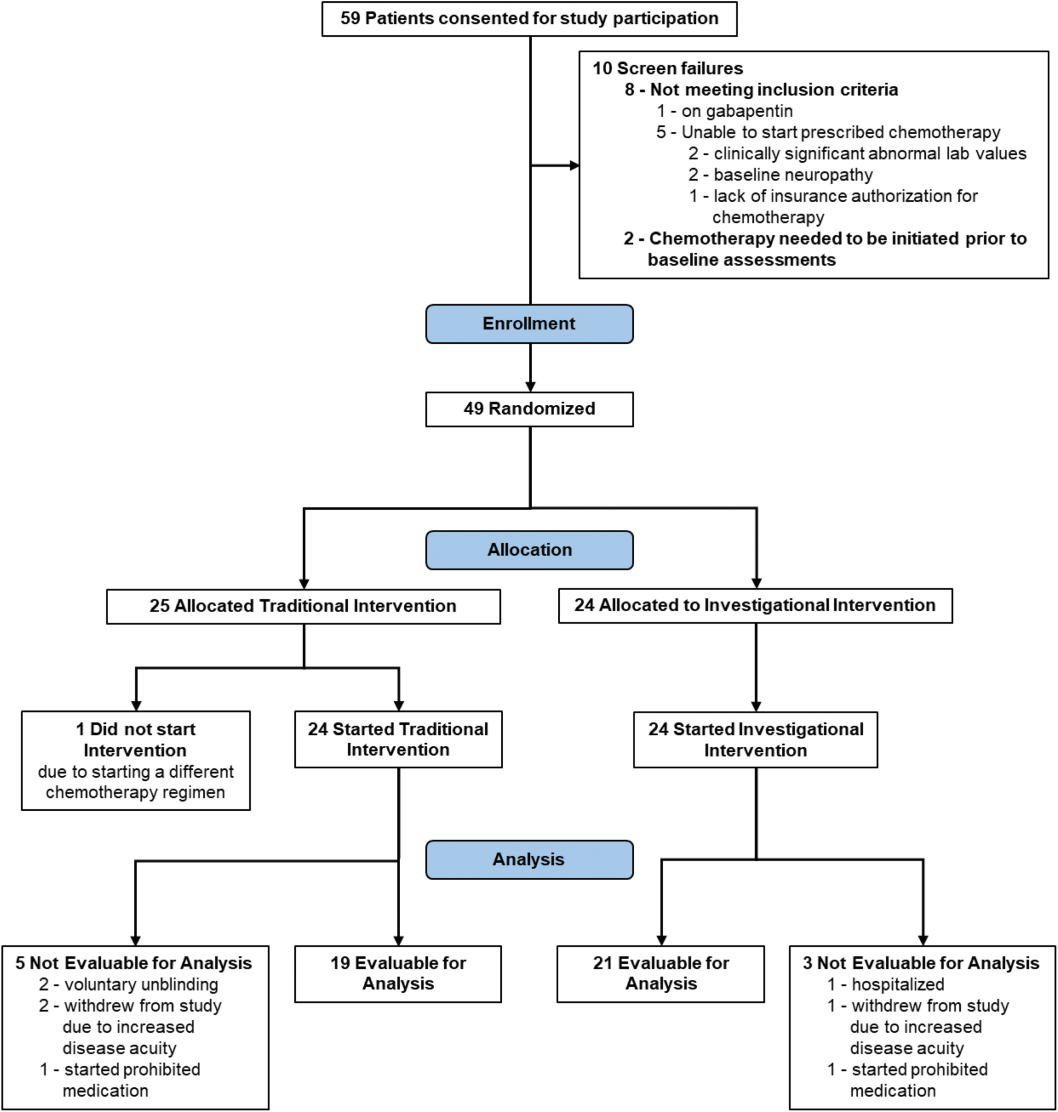
CONSORT diagram of patient flow through study.

**Figure 2.**
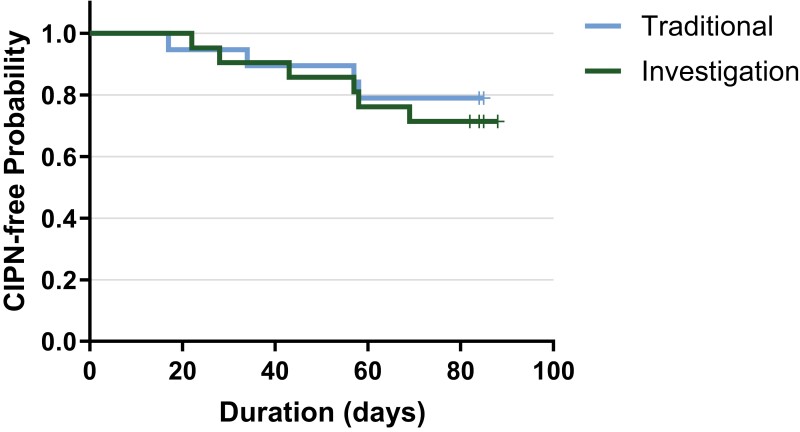
Time to onset. Mean time-to-event of onset of CIPN was 75.8 (90% CI: 68.5, 83.2) days in the traditional group and 76.0 (90% CI: 68.5, 83.6) days in the investigational group, *P* = .614. Fifteen of 19 patients (78.9%) in traditional group and 15/21 (71.4%) in investigational group were censored in the analysis as they did not develop CIPN while on study.

**Figure 3.**
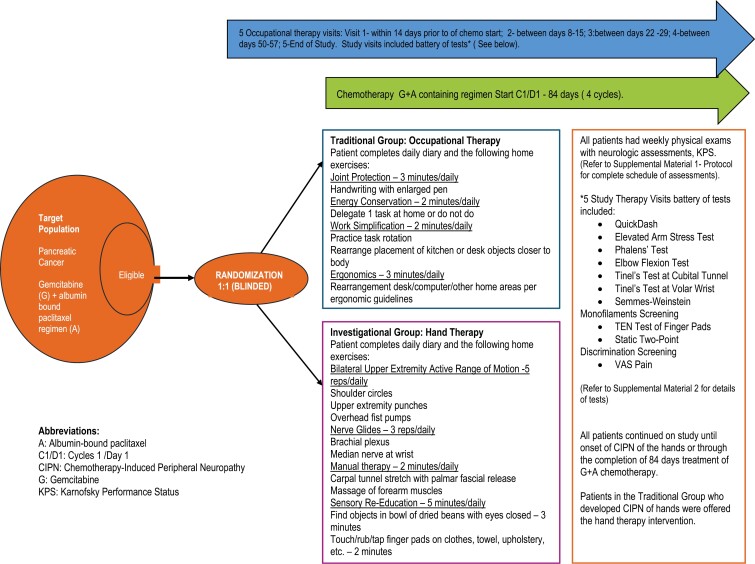
Study diagram.

## Additional details of endpoints or study design

### Patient selection

#### Inclusion criteria

1. Patients with adenocarcinoma of the pancreas who will receive chemotherapy with albumin-bound paclitaxel plus gemcitabine containing combination.
2. Patients who have no evidence of past or current peripheral neuropathy of the hands.
3. Age 18 years or older.
4. Able to sit for minimum of 30 minutes for hand therapy sessions.

#### Exclusion criteria

1. Patients who have received at least one dose of chemotherapy with albumin-bound paclitaxel plus gemcitabine containing combination during the past 6 months.
2. Patients taking duloxetine or gabapentin.
3. History of peripheral neuropathy of the hands due to chemotherapy or other causes.
4. Inability to sit for minimum of 30 minutes.
5. Documented cognitive problems limiting ability to participate in hand therapy.

#### Treatment plan

The schedule of study procedures included a consent visit/screening period within 14 days of the start of the study; demographic and medical history; documentation of medications, and physical examination. The physical examination included weight, a brief neurological examination of the upper extremities including reflexes, strength, and neurological sensory testing. The Common Terminology Criteria for Adverse Events, version 4 (CTCAE-4.0) was utilized to grade CIPN of hands and the patient performance status was evaluated using the Karnofsky Performance Status (KPS).

Patients were randomly assigned to one of the two intervention groups (Traditional or Investigational) by the study data specialist using a random scramble formula in EXCEL after the patient signed consent. As this was a double-blind, randomized trial, the identity of the intervention was to remain unknown to the patients and Investigators until the end of the study once the database was locked. Only the study coordinator and occupational therapists were aware of the assigned intervention group (Figures 1, 2, and 3).

### Traditional group

Traditional group subjects were instructed in joint protection, energy conservation, work simplification, and ergonomics, and they were provided with a catalogue of occupational therapy assistive devices. This traditional occupational therapy program is based on core domain features identifying scope of practice of the profession.

### Investigational group

Investigational group subjects were instructed in bilateral upper extremity active range of motion (AROM), brachial plexus nerve gliding exercises, manual therapy of the forearm and hand, and sensory-re- education techniques. This evidence-based intervention is intended to maximize peripheral nerve axoplasmic flow resulting in enhanced nerve transmission.

Five occupational therapy visits were provided for each patient (across both groups) during their routine clinic visits. Routine physical examinations including neurological assessment were performed weekly. The assessments performed by an occupational therapist at each study therapy visit included: the QuickDASH outcome measure; a brief battery of upper extremity provocative testing consisting of the Elevated Arm Stress Test (EAST), Phalen’s Test, Tinel Test at the Volar Wrist, Elbow Flexion Test, Tinel Test at the Cubital Tunnel; pressure threshold screening; Ten Test screening, static two-point discrimination screening; and the Visual Analog Scale (VAS) for pain. All patients were asked to complete a daily patient diary to ensure compliance with prescribed OT intervention. The diary was collected and reviewed by study staff weekly. Patients were compensated modestly for their time and effort in participating in the study and completing patient diaries.

#### Duration

Patients participated until the time of onset of CIPN of the hands or through 84 days of treatment if no symptoms of CIPN. At time of initial onset of any grade of CIPN of the hands, the patient will end participation in the study. For study purposes, the presence of CIPN was defined as perceived sensory changes in the hands including numbness or tingling persisting > 1 hour, or evidence of motor neuropathy on medical examination. The patient was able to continue on study if CIPN occurred only in the feet. Duration of overall study was 12 months.

## Drug Information

**Table:** 

	Arm 1	Arm 2
Generic/working name	Traditional occupational therapy	Investigational hand therapy
Schedule of administration	Traditional occupational therapy was completed at 5 visits as follows: Visit #1: between consent and day 1 of chemotherapy Visit #2: between days 8 and 15 of chemotherapy Visit #3: between days 22 and 29 of chemotherapy Visit #4:between days 50 and 57 of chemotherapy Visit #5: at end of study	Traditional occupational therapy was completed at 5 visits as follows: Visit #1: between consent and day 1 of chemotherapy Visit #2: between days 8 and 15 of chemotherapy Visit #3: between days 22 and 29 of chemotherapy Visit #4: between days 50 and 57 of chemotherapy Visit #5: at end of study

## Patient Characteristics

**Table:** 

Patient characteristics	Cohort name: traditional group
Number of patients, male	13
Number of patients, female	12
Stage	Stage was not collected for this study 12 patients did not have metastatic disease 13 patients had metastatic disease
Age: Median (range)	67 (38-84) years
Number of prior systemic therapies: median (range)	1 patient with 1 prior systemic therapy
Performance status: ECOG	0: 12 1: 11 2: 2 3: 0 4: 0
Cancer types or histologic subtypes	Adenocarcinoma of the pancreas, 25

## Patient Characteristics

**Table:** 

Patient characteristics	Cohort name: investigational group
Number of patients, male	12
Number of patients, female	12
Stage	Stage was not collected for this study 6 patients did not have metastatic disease 18 patients had metastatic disease
Age: median (range)	69 (42-83) years
Number of prior systemic therapies: median (range)	1 patient with 1 prior systemic therapy
Performance status: ECOG	0: 11 1: 13 2: 0 3: 0 4: 0
Cancer types or histologic subtypes	Adenocarcinoma of the pancreas, 24

## Primary Assessment Method: Traditional Group

**Table:** 

Number of patients screened	Total of 59 across both groups before randomization
Number of patients enrolled	25
Number of patients evaluable for toxicity	24
Number of patients evaluated for efficacy	19
Evaluation method	The number and proportion of patients without CIPN (any grade) of the hands at the completion of 84 days of chemotherapy treatment
Response duration	15/19 (78.8%) patients completed 84 days of treatment without neuropathy
Duration of treatment	84 days or until CIPN developed
Outcome notes	In the traditional group, 15/19 patients (78.8%) completed 84 days of treatment without neuropathy. Overall mean compliance with home prescribed occupational therapy based on patient completed diary was 92.2% in 16/19 patients that returned their patient diaries. Four of 19 patients (21.1%) in traditional group developed CIPN, *P* = .721. Cumulative doses of albumin-bound paclitaxel and cisplatin per patient of those who developed CIPN are listed in Tables 1 and 2. Patient characteristics, disease states, treatment dates, number of days on study, and time to onset of CIPN are detailed in Supplementary Table S1. Results of a Kaplan-Meier analysis showed those in Traditional Group with a mean time-to-event of 75.8 days (90% CI: 68.5, 83.2). Fifteen of 19 (78.9%) in the traditional group were censored in the analysis as they did not develop CIPN while on study (Figure 2).

## Primary Assessment Method: Investigational Group

**Table:** 

Number of patients screened	Total of 59 across both groups before randomization
Number of patients enrolled	24
Number of patients evaluable for toxicity	24
Number of patients evaluated for efficacy	21
Evaluation method	The number and proportion of patients without CIPN (any grade) of the hands at the completion of 84 days of chemotherapy treatment
Response duration	15/21 (71.4%) Patients completed 84 days of treatment without neuropathy
Duration of treatment	84 days or until CIPN developed
Outcome notes	In the investigational group, 15/21 patients (71.4%) completed 84 days without neuropathy. Overall mean compliance with home prescribed occupational therapy based on patient completed diary was 87.9% in 20/21 patients that returned their patient diaries. Six of 21 patients (28.6%) in Investigational Group developed CIPN, *P* = .721. Cumulative doses of albumin-bound paclitaxel and cisplatin per patient of those who developed CIPN are listed in Tables 1 and 2. Patient characteristics, disease states, treatment dates, number of days on study, and time to onset of CIPN are detailed in Supplementary Table S1. Results of a Kaplan-Meier analysis showed those in investigational group with a mean time-to-event of 76.0 days (90% CI: 68.5, 83.6), *P* = .614. Fifteen of 21 (71.4%) in the investigational group were censored in the analysis as they did not develop CIPN while on study (Figure 2).

## Secondary Assessment Method: Provocative Testing of Upper Extremities

**Table:** 

Evaluation method	Ten provocative tests of the upper extremities predict patients at risk for CIPN
Outcome notes	Ten provocative tests of the upper extremities were administered at each session to each patient (1900 tests). The tests were the Elevated Arm Stress Test (EAST), Phalen’s Test, Tinel Test at the Volar Wrist, Elbow Flexion Test, and Tinel Test at the Cubital Tunnel, each done on both arms. Of these, only 22 of the 1900 tests (1.2%) yielded positive results. Refer to Supplementary Tables S2-S11.

## Secondary Assessment Method: TEN TEST and the QuickDASH score correlation with CIPN grade

**Table:** 

Evaluation method	Correlation of the TEN TEST and the QuickDASH score to grades peripheral neuropathy (NCT-CTC AE V4.0)
Outcome notes	Spearman correlation coefficients between the QuickDASH and Ten Test scores to grades of peripheral neuropathy are detailed in Tables 3 and 4. There was no significant correlation between the Ten Test for finger pad sensation and the QuickDASH functional outcome measure and the risk of developing CIPN. There was no correlation in the risk of developing CIPN with age, sex, BMI, disease state, or KPS in this small sample of patients

## Assessment, Analysis, and Discussion

**Table:** 

Completion	Study completed
Investigator’s assessmen**t**	Correlative endpoints not met but clinical activity observed. While there was no difference between the type of intervention, early adaption of occupational therapy may prevent the development of early onset CIPN in most individuals receiving neuropathy inducing chemotherapy

The primary objective of this randomized double-blind clinical trial was to determine if a hand therapy intervention targeting the sensory system could prevent or delay the time to onset of any grade of CIPN of the hands more effectively than the traditional occupational therapy intervention in patients with newly diagnosed metastatic pancreatic cancer receiving gemcitabine and albumin-bound paclitaxel chemotherapy. This evidence-based hand therapy intervention used in this study was extrapolated from research in hand surgery and is the basis of sensory rehabilitation for people with upper extremity injuries or problems. The Investigational intervention consisted of pain-free, pleasant active range of motion (AROM), nerve and tendon glides; manual therapy targeting areas of potential nerve entrapment or vulnerability; and sensory rehabilitation.^1^

**Table 1.** Total cumulative doses of albumin bound paclitaxel and cisplatin per patient of those who developed CIPN (*n* = 10).

Patient no.	Sex	Age	Metastatic disease y/n	CIPN onset day	Cum dose albumin bound paclitaxel (mg)	Cum dose cisplatin (mg)
Investigational hand therapy arm *N* = 21 6/21 (29%)						
2	F	52	y	47	1575	318
4	F	77	y	28	657	132
17	M	54	y	67	1647	331
36	M	72	y	43	1098	174
45	M	71	y	57	1326	318
47	M	79	y	56	1488	300
Occupational therapy arm *N* = 19 4/19 (21%)						
12	M	70	y	17	522	104
16	F	69	n	57	1215	246
28	M	64	y	34	1200	256
53	F	66	y	21	380	76

Abbreviations: Cum, cumulative; CIPN, chemotherapy-induced peripheral neuropathy; n, no; y, yes.

**Table 2.** Summary of cumulative doses of albumin bound paclitaxel and cisplatin in all patients (*n* = 40).

Neuropathy		Albumin bound paclitaxel	Cisplatin
No	Mean	1749.53	350.60
	Median	1792.00	364.00
	Minimum	988	0
	Maximum	2520	504
	Range	1532	504
	Number	30	30
	Standard Deviation	344.129	97.901
Yes	Mean	1110.80	225.50
	Median	1207.50	251.00
	Minimum	380	76
	Maximum	1647	331
	Range	1267	255
	Number	10	10
	Standard Deviation	446.872	96.408
Total	Mean	1589.85	319.33
	Median	1600.00	330.50
	Minimum	380	0
	Maximum	2520	504
	Range	2140	504
	Number	40	40
	Standard Deviation	461.086	110.822

**Table 3:** QuickDASH scores.

Arm	Visit	Mean	Standard deviation
Traditional	1	7.06	8.26
	2	7.49	10.02
	3	6.10	8.80
	4	7.84	11.72
	5	6.20	10.05
Investigational	1	7.36	10.31
	2	6.06	9.27
	3	6.49	9.43
	4	8.01	10.51
	5	5.30	7.95

Refer to Supplement 2. The QuickDASH is a shorter version of the DASH Outcome Measure, which is a 30-item, self-report questionnaire that is designed to measure symptoms and physical function in people with any of a variety of musculoskeletal disorders involving the upper limb. It is often used to monitor changes in function and symptoms over time.

**Table 4.** QuickDASH and Ten Test Correlations.

			QuickDASH
Spearman’s rho	Ten test—R Thb	Correlation coefficient	−0.077
		Sig. (2-tailed)	0.291
		*N*	188
	Ten test—L Thb	Correlation coefficient	−0.106
		Sig. (2-tailed)	0.148
		*N*	188
	Ten rest—R SF	Correlation coefficient	−0.127
		Sig. (2-tailed)	0.085
		*N*	186
	Ten rest—L SF	Correlation coefficient	−0.089
		Sig. (2-tailed)	0.228
		*N*	187

The QuickDASH is a shorter version of the DASH outcome measure, which is a 30-item, self-report questionnaire that is designed to measure symptoms and physical function in people with any of a variety of musculoskeletal disorders involving the upper limb. It is often used to monitor changes in function and symptoms over time.

Abbrevations: L, left; N, number; R, right; SF, small finger; Sig, significance; Thb, thumb.

There were no statistically significant differences in the time to onset of CIPN (*P* = .721) or the home prescribed therapy compliance (*P* = .488) between the 2 interventions. However, it is encouraging that 75% of patients in this small feasibility study did not develop CIPN by day 84 (12 weeks) of chemotherapy. This low incidence of CIPN raises the question whether any hand therapy would delay the onset of CIPN. In a recent study, 91.6% of women with breast cancer receiving nab-paclitaxel (295 patients) reported CIPN by 6 weeks.^2^ One possible explanation is that clinical care and coaching by the occupational therapists might have decreased perceived sensation changes. Additionally, study participation and attention to sensory changes might have had an impact on symptom reporting.

Several interesting issues warrant discussion. The median time to onset of any grade of CIPN in our study was 76 days compared to 71 days observed in the IMPACT Trial^3^ (Von Hoff personal communication). Only the median time to onset of grade 3 CIPN, not including grades 1 and 2 of 140 days was reported in the IMPACT trial.^4^ In the present study, thirty nine of 40 patients received an additional neurotoxic agent, cisplatin, which was added to gemcitabine and albumin-bound paclitaxel and did not appear to increase time to onset of CIPN. However, no direct comparison can be made with the gemcitabine and albumin-bound paclitaxel administered in the IMPACT trial. Also, cisplatin has been associated with neuropathic pain,^5^ yet no patients reported painful neuropathy of the hands at time of onset, only grade 1 sensation changes.

In this small patient sample, we found no correlation between the risk of developing CIPN and age, sex, BMI, disease state, or KPS. Physical examinations and occupational therapy assessments did not predict the reported onset of sensory CIPN, and no patients experienced motor neuropathy.

This is the first study to report time to onset of grade 1 CIPN (paresthesia—CTCAE v 4), in patients receiving GA containing chemotherapy. Additionally, this is the first report on the use of the Ten Test for screening of sensory status of the hand in a cancer population.^6^ This is the first study to report on a range of upper extremity measures, including provocative and positional upper extremity tests, for CIPN. We chose this battery as we found it to be clinically relevant for symptomatic CIPN patients, but this battery did not yield meaningful findings in this prevention study. We suggest it may be more useful in treatment studies and recommend further research in this area.

In our clinical experience, it is not uncommon for our CIPN patients to remark with surprise that they experience sensory improvement during the treatment process, and we have seen improved Ten Test (sensory) results following treatment. If CIPN symptoms are attributable only to neurotoxic tissue damage,^7^ then why do active movement, nerve and tendon gliding, and sensory stimulation provide such immediate relief? We suggest that CIPN symptoms caused by chemotherapy toxicity may be compounded by subclinical edema (ie, edema that is not measurable and not visible). Subclinical edema is associated with inactivity, fatigue, and poor posture, all of which are common in cancer populations. It is well-known that active movement, nerve and tendon gliding, and sensory stimulation reduce subclinical edema by promoting lymphatic flow.^8^ We have not seen research addressing the impact of subclinical edema on CIPN symptoms. Our clinical experience suggests this intriguing question warrants further research.

## Limitations

The main limitation of this study was its exclusive focus on the hands, omitting neurological changes in the feet or other body parts. Additionally, the absence of a control group without hand therapy and the small sample size limited the study. Recruitment over four years at a single, high-volume pancreatic cancer center was challenging. We speculate that the emotional and symptom burdens of newly diagnosed pancreatic cancer patients, along with the urgency to start treatment, reduced their interest in participating in a supportive care prevention study, although this was not specifically examined.

A battery of upper extremity assessments was used to monitor for the development of CIPN symptoms (Supplement 2). This battery is considered appropriate and relevant in traditional rehabilitation for patients with other sensory problems such as nerve entrapment or nerve laceration.^9-12^ Patients continued on study only as long as they were asymptomatic. Study participation ended if/when they became symptomatic. There were very few symptom-related findings, and these occurred only near or at the time that patients’ participation ended, thereby limiting the existence of symptom-related data. More clinical findings and more data would have been identified in a study that was longer in duration. Given the duration of 84 days and low incidence of CIPN, a more valuable design would involve a larger sample size or a longer timeframe. However, studying this in pancreatic cancer patients presents challenges, as they may discontinue treatment due to disease progression or treatment-related toxicities after 3 months, necessitating changes in treatment.

The investigational home program was shortened to accommodate lower energy levels or reduced tolerance anticipated in our study population due to their clinical condition. Exploring a program with increased repetitions and longer engagement times could potentially have a more beneficial impact and should be considered for further research.

## Supplementary material

Supplementary material is available at *The Oncologist* online.

oyae285_suppl_Supplementary_Tables

## Data Availability

The data that support the findings of this study are available on request from the corresponding author. Data availability is subject to institutional approval.
