# Supplementary material for: Hand therapy interventions for the prevention of chemotherapy-induced peripheral neuropathy of the hands in patients with pancreatic cancer
Source: Oncologist. 2024 Oct 22;29(12):1095–e1778. doi: 10.1093/oncolo/oyae285 (PMC11630791; doi:10.1093/oncolo/oyae285)
Supplement: oyae285_suppl_Supplementary_Tables [file oyae285_suppl_supplementary_tables.pdf]

**Supplemental Materials**

1. Protocol
2. Battery of Upper Extremity Testing

**Supplemental Tables**

Supplemental Table 1: Patient Characteristics, Disease State, Treatment Dates, Number of Days on Study, Time to Onset of CIPN
Supplemental Table 2: Elevated Arm Stress Test Right Arm
Supplemental Table 3: Elevated Arm Stress Test Left Arm
Supplemental Table 4: Phalen's Test Right Arm
Supplemental Table 5: Phalen's Test Left Arm
Supplemental Table 6: Elbow Flexion Test Right Arm
Supplemental Table 7: Elbow Flexion Test Left Arm
Supplemental Table 8: Tinel Test Right Volar Wrist
Supplemental Table 9: Tinel Test Left Volar Wrist
Supplemental Table 10: Tinel Test Right Cubital Tunnel
Supplemental Table 11: Tinel Test Left Cubital Tunnel

**Supplemental Material 1 – Protocol**

See separate file.

**Supplemental Material 2: Battery of Upper Extremity Testing**

**QUICKDASH**
QuickDASH [1,2]

The QuickDASH is a shorter version of the DASH Outcome Measure, which is a 30-item, self-report questionnaire that is designed to measure symptoms and physical function in people with any of a variety of musculoskeletal disorders involving the upper limb. It is often used to monitor changes in function and symptoms over time. The shorter version is valid, reliable and responsive and is popular for use in clinical programs and in research. A variety of translations are available.

**ELEVATED ARM STRESS TEST (EAST)**
Elevated Arm Stress Test (EAST) [3-6]

Also called Roos' test, the EAST is performed by holding the arms in a position of 90 degrees of abduction and external rotation ("stick-up position"). The patient then opens and closes the hands slowly for a period of 3 minutes. Reproduction of symptoms in the entire extremity or rapid fatigue of the extremity constitutes a positive test result. Patients with thoracic outlet syndrome (TOS) typically cannot complete this test. Many authors describe this as the most reliable test for TOS. The test is of the most diagnostic benefit when symptoms occur rapidly after elevation of the arm. One study showed reproduction of symptoms in 94% of patients with neurogenic TOS. This maneuver can reproduce symptoms of other pathologies, including carpal tunnel syndrome, cubital tunnel syndrome, and rotator cuff syndrome.

**PHALEN'S TEST**
Phalen's Test [7,8]

Phalen's test involves passive flexion of the wrist for 15 to 60 seconds. A positive test produces numbness and tingling in the distribution of the median nerve. Researchers have reported that this test is one of two clinical tests with the highest overall accuracy in testing for median nerve compression at the wrist.

**TINEL'S TEST AT THE VOLAR WRIST**
Tinel's Test at the Volar Wrist [9]

The examiner taps over the carpal tunnel. Reproduction of paresthesias in the distribution of the median nerve is a positive response.

## ELBOW FLEXION TEST

Elbow Flexion Test [10]

The Elbow Flexion test is one of the most sensitive and specific tests for identifying ulnar nerve compression at the cubital tunnel. With the shoulder in neutral position, manual pressure is applied to the ulnar nerve in the cubital tunnel as the elbow is placed in terminal flexion with the forearm supported in supination. Maintain the wrist in neutral. Hold the position for 60 seconds.

## TINEL'S TEST AT THE CUBITAL TUNNEL

Tinel's Test at the Cubital Tunnel [11]

With the patient's elbow fully flexed and the wrist held in neutral position, the examiner taps over the ulnar nerve with the cubital tunnel. Reproduction of paresthesias in the distribution of the ulnar nerve is a positive response.

## SEMMES WEINSTEIN MONOFILAMENTS TEST FOR PRESSURE THRESHOLD SCREENING

Semmes Weinstein Monofilaments for Pressure Threshold Screening [12-14]

When calibrated and applied correctly, this test is a valid test for determining sensibility detection thresholds. Studies have clearly demonstrated their ability to accurately detect intended clinical conditions. Used in standard consistent protocols, the monofilament test is able to compare patient data in individual and multicenter studies and is providing information regarding peripheral nerve changes with treatment not previously available with less sensitive and uncontrolled instruments. When calibrated correctly, it is one of the few, if not the only, sensibility measurement instrument that approaches requirements for an objective test.

The hand screen utilizes 5 monofilaments. Test sites specific to the median nerve are the tip of the thumb, index, and proximal index. Test sites specific to the ulnar nerve are the distal little finger, proximal phalanx, and ulnar base of the palm. The test site specific for the radial nerve is the dorsal aspect of the thumb web space. The examiner retests a nonresponsive test site at least three times to ensure a monofilament is not detected at that site.

## TEN TEST

Ten Test [15]

In the Ten Test of finger pad light sensation, the patient develops a ratio between normal light moving touch and diminished moving touch. Subsequent determinations can detect serial changes. The ratios obtained can be compared with a standard scale of sensibility with a high degree of validity and reliability. The interexaminer and

intraexaminer results obtained are reliable and repeatable. Simplicity and dependability recommend this test for use in a busy clinical setting.

## STATIC TWO-POINT DISCRIMINATION SCREENING

Static Two-Point Discrimination Screening [16-20]

Two-point discrimination is a classic test of sensibility used by hand surgeons over several decades. The test is believed by many to be a test of innervation density. Some think two-point discrimination a good predictor of patient function and manipulation. Two-point discrimination testing is most accurate at the fingertips. Normal two-point discrimination is considered less than 6 mm, fair is 6 to 10 mm, and poor is 11 to 15 mm.

## VISUAL ANALOG PAIN SCALE (VAS)

Visual Analog Pain Scale [21,22]

The Visual Analog Scale (VAS) for pain is used to measure quantity of pain. A 10-cm line has 0 as no pain and 10 as worst pain imaginable. High test-retest reliability has been demonstrated. There is a high correlation between VAS and the numeric pain rating scale and a verbal description of pain.

The VAS is well described in research, and has been shown to be a valid, reliable and sensitive clinical measure of pain that is amenable to statistical analysis. It is simple to use. VAS showed significant positive correlation with numeric rating scales and the faces pain scale. Previous studies have shown reliability, validity and clinical sensitivity of the VAS as a measure of intensity of subjective pain.

## Supplemental Table 1: Evaluable Patient Characteristics, Disease State, Treatment Dates, Number of Days on Study, Time to Onset of CIPN (N=40)

| Subject Number | Race/Sex/Ethnicity | Weight (kg) | Height (cm) | BMI (kg/m2) | Age | Disease State | Treatment Arm | Study Treatment Start Date | Study Treatment Stop Date | Number of Days on Study | Onset of CIPN |
|---|---|---|---|---|---|---|---|---|---|---|---|
| 1 | W/M/NH | 124.6 | 183.7 | 36.9 | 38 | Non-Metastatic | Traditional | 8/4/16 | 10/27/16 | 84 | |
| **2** [a] | **W/F/NH** | **96** | **167.7** | **34.1** | 52 | **Metastatic** | **Investigational** | **8/16/16** | **10/7/16** | **58** | **10/1/2016 - Day 47** |
| **4** | **W/F/NH** | **69.2** | **160.5** | **26.9** | 77 | **Metastatic** | **Investigational** | **8/25/16** | **9/22/16** | **28** | **9/21/2016 - Day 28** |
| 5 | W/F/NH | 71.5 | 172 | 24.2 | 67 | Metastatic | Traditional | 8/24/16 | 11/16/16 | 84 | |
| 10 | W/M/NH | 99.6 | 188 | 28.2 | 58 | Metastatic | Investigational | 1/17/17 | 4/11/17 | 84 | |
| 11 | W/M/NH | 75.4 | 180 | 23.3 | 66 | Metastatic | Traditional | 4/12/17 | 7/5/17 | 84 | |
| **12** | **W/M/NH** | **81.2** | **193.5** | **21.7** | 70 | **Metastatic** | **Traditional** | **5/25/17** | **6/11/17** | **17** | **6/11/2017 - Day 17** |
| 13 | W/F/NH | 52.7 | 154.9 | 22 | 71 | Metastatic | Investigational | 7/5/17 | 9/27/17 | 84 | |
| 14 | W/M/NH | 82.9 | 175.3 | 27 | 61 | Metastatic | Investigational | 7/10/17 | 10/2/17 | 84 | |
| 15 | W/F/NH | 67.9 | 179 | 21.2 | 70 | Non-Metastatic | Traditional | 7/26/17 | 10/18/17 | 84 | |
| **16** | **W/F/NH** | **59.1** | **162.1** | **22.5** | 77 | Non-Metastatic | **Traditional** | **8/15/17** | **10/10/17** | **58** | **10/10/2017 -D ay 57** |
| **17** | **W/M/NH** | **76.6** | **180.3** | **23.6** | 55 | **Metastatic** | **Investigational** | **10/4/17** | **12/12/17** | **69** | **12/9/2017 - Day 67** |
| 18 | W/F/NH | 62.7 | 165.1 | 23 | 68 | Non-Metastatic | Traditional | 10/11/17 | 1/3/18 | 84 | |
| 19 | W/M/NH | 86.5 | 178.9 | 27 | 83 | Non-Metastatic | Investigational | 11/2/17 | 1/26/18 | 85 | |
| 20 | W/F/NH | 60.4 | 155 | 25.1 | 65 | Metastatic | Traditional | 12/6/17 | 2/28/18 | 85 | |
| 21 | W/F/NH | 51 | 167.6 | 18.2 | 64 | Metastatic | Traditional | 12/8/17 | 3/8/18 | 85 | |
| 22 | W/M/NH | 84.9 | 180.3 | 26.1 | 42 | Metastatic | Investigational | 2/8/18 | 5/2/18 | 84 | |
| 23 | W/M/NH | 69.3 | 183.3 | 20.6 | 70 | Metastatic | Investigational | 2/8/18 | 5/7/18 | 88 | |
| 24 | W/F/NH | 65.2 | 154.9 | 27.2 | 61 | Metastatic | Traditional | 2/7/18 | 5/3/18 | 84 | |
| 25 | W/M/NH | 96.5 | 177.8 | 30.6 | 64 | Non-Metastatic | Traditional | 2/7/18 | 5/7/18 | 84 | |
| **28** | **W/M/NH** | **125.1** | **188** | **35.1** | 64 | **Metastatic** | **Traditional** | **7/18/18** | **8/21/18** | **34** | **8/21/2018 - Day 34** |

| Subject Number | Race/Sex/Ethnicity | Weight (kg) | Height (cm) | BMI (kg/m2) | Age | Disease State | Treatment Arm | Study Treatment Start Date | Study Treatment Stop Date | Number of Days on Study | Onset of CIPN |
|---|---|---|---|---|---|---|---|---|---|---|---|
| 31 | W/M/NH | 88 | 175.3 | 28.6 | 79 | Non-Metastatic | Traditional | 9/17/18 | 12/10/18 | 84 | |
| 34 | W/M/NH | 78.4 | 188.1 | 22.2 | 84 | Non-Metastatic | Traditional | 10/31/18 | 1/21/19 | 84 | |
| 35 [b] | W/F/NH | 50.4 | 152.4 | 21.7 | 73 | Non-Metastatic | Investigational | 11/1/18 | 1/31/19 | 84 | |
| **36** | **W/M/NH** | **104** | **182.9** | **31.1** | 72 | Non-Metastatic | **Investigational** | **12/4/18** | **1/16/19** | **43** | **1/16/2019 - Day 43** |
| 37 | W/M/NH | 86.8 | 175.3 | 28.2 | 69 | Non-Metastatic | Traditional | 12/18/18 | 3/12/19 | 84 | |
| 39 | W/F/NH | 72.6 | 170.2 | 25.1 | 61 | Metastatic | Investigational | 4/1/19 | 6/24/19 | 84 | |
| 40 | W/M/NH | 101.4 | 180.3 | 31.2 | 73 | Non-Metastatic | Investigational | 4/25/19 | 7/18/19 | 84 | |
| 41 | W/M/NH | 73.3 | 176.5 | 23.5 | 78 | Non-Metastatic | Investigational | 5/30/19 | 8/23/19 | 82 | |
| 42 | W/M/NH | 73.5 | 172.7 | 24.6 | 63 | Metastatic | Traditional | 6/4/19 | 8/28/19 | 84 | |
| **45** | **W/M/NH** | **83.2** | **190.5** | **22.9** | 72 | Non-Metastatic | **Traditional** | **7/25/19** | **9/19/19** | **57** | **9/19/2019 - Day 57** |
| 46 | W/F/NH | 57.5 | 165.1 | 21.1 | 65 | Non-Metastatic | Investigational | 8/21/19 | 11/13/19 | 84 | |
| **47** | **A/M/NH** | **76.7** | **180.3** | **23.6** | 80 | **Metastatic** | **Investigational** | **9/26/19** | **11/26/19** | **57** | **11/20/2019 - Day 56** |
| 48 | W/F/NH | 72.3 | 172.7 | 24.2 | 70 | Metastatic | Investigational | 9/24/19 | 12/16/19 | 84 | |
| 49 | W/M/NH | 86 | 188 | 24.3 | 61 | Metastatic | Investigational | 10/14/19 | 1/7/20 | 84 | |
| 51 | W/M/NH | 85.6 | 177.8 | 27.1 | 79 | Non-Metastatic | Traditional | 12/19/19 | 3/12/20 | 84 | |
| 52 | W/M/NH | 56.3 | 172.7 | 18.9 | 75 | Metastatic | Investigational | 1/14/20 | 4/7/20 | 84 | |
| **53** | **W/F/H** | **56.4** | **149.9** | **25.1** | 67 | **Metastatic** | **Investigational** | **1/15/20** | **2/5/20** | **22** | **2/4/2020 - Day 21** |
| 54 | W/F/NH | 69.9 | 167.6 | 24.9 | 62 | Metastatic | Traditional | 1/16/20 | 4/21/20 | 84 | |
| 55 | W/F/H | 87.6 | 162.6 | 33.1 | 56 | Metastatic | Investigational | 6/22/20 | 9/15/20 | 84 | |

Abbreviations: CIPN=Chemotherapy Induced Peripheral Neuropathy, W=White, A=Asian, M=Male, F=Female, H=Hispanic, NH=Non-Hispanic
[a] Patient was a screen failure due to abnormal lab value (elevated bilirubin). Once resolved, the patient was reconsented, assigned a new subject number, and enrolled into study.
[b] Patient received Gemcitabine+Albumin-Bound Paclitaxel chemotherapy, all others received Gemcitabine+Albumin-Bound Paclitaxel+Cisplatin
Note: Patients who developed CIPN are in bold.

**Supplemental Table 2: Elevated Arm Stress Test (EAST) Right Arm [3-6]**

| Visit | Group | Negative | Positive | Missing/Not Tested | Total |
|---|---|---|---|---|---|
| 1 | Traditional | 19 (100) | 0 | 0 | 19 |
| | Investigation | 20 (95.2) | 0 | 1 (4.8) | 21 |
| 2 | Traditional | 19 (100) | 0 | 0 | 19 |
| | Investigation | 21 (100) | 0 | 0 | 21 |
| 3 | Traditional | 19 (100) | 0 | 0 | 19 |
| | Investigation | 20 (95.2) | 1 (4.8) | 0 | 21 |
| 4 | Traditional | 18 (100) | 0 | 0 | 18 |
| | Investigation | 18 (100) | 0 | 1 (5.6) | 18 |
| 5 | Traditional | 17 (100) | 0 | 0 | 17 |
| | Investigation | 15 (93.8) | 0 | 1 (6.3) | 16 |
| Total | Traditional | 92 (100) | 0 | 0 | 92 |
| | Investigation | 94 (95.9) | 1 (1.0) | 3 (3.1) | 98 |

QuickDASH [1,2] - Refer to Supplemental Material 2

**Supplemental Table 3: Elevated Arm Stress Test (EAST) Left Arm [3-6]**

| Visit | Group | Negative | Positive | Missing/Not Tested | Total |
|---|---|---|---|---|---|
| 1 | Traditional | 19 (100) | 0 | 0 | 19 |
| | Investigation | 21 (100) | 0 | 0 | 21 |
| 2 | Traditional | 19 (100) | 0 | 0 | 19 |
| | Investigation | 21 (100) | 0 | 0 | 21 |
| 3 | Traditional | 19 (100) | 0 | 0 | 19 |
| | Investigation | 21 (100) | 0 | 0 | 21 |
| 4 | Traditional | 18 (100) | 0 | 0 | 18 |
| | Investigation | 18 (94.7) | 0 | 1 (5.3) | 19 |
| 5 | Traditional | 17 (100) | 0 | 0 | 17 |
| | Investigation | 15 (93.8) | 0 | 1 (6.3) | 16 |
| Total | Traditional | 92 (100) | 0 | 0 | 92 |
| | Investigation | 96 (98.0) | 0 | 2 (2.0) | 98 |

**Supplemental Table 4: Phalen's Test Right Arm** [7,8]

| Visit | Group | Negative | Positive | Missing/Not Tested | Total |
|---|---|---|---|---|---|
| 1 | Traditional | 19 (100) | 0 | 0 | 19 |
| | Investigation | 21 (100) | 0 | 0 | 21 |
| 2 | Traditional | 19 (100) | 0 | 0 | 19 |
| | Investigation | 21 (100) | 0 | 0 | 21 |
| 3 | Traditional | 19 (100) | 0 | 0 | 19 |
| | Investigation | 20 (95.2) | 1 (4.8) | 0 | 21 |
| 4 | Traditional | 18 (100) | 0 | 0 | 18 |
| | Investigation | 17 (89.4) | 1 (5.3) | 1 (5.3) | 19 |
| 5 | Traditional | 15 (88.2) | 2 (11.7) | 0 | 17 |
| | Investigation | 15 (93.8) | 0 | 1 (6.3) | 16 |
| Total | Traditional | 90 (97.8) | 2 (2.2) | 0 | 92 |
| | Investigation | 94 (95.9) | 2 (2.0) | 2 (2.0) | 98 |

**Supplemental Table 5: Phalen's Test Left Arm** [7,8]

| Visit | Group | Negative | Positive | Missing/Not Tested | Total |
|---|---|---|---|---|---|
| 1 | Traditional | 19 (100) | 0 | 0 | 19 |
| | Investigation | 21 (100) | 0 | 0 | 21 |
| 2 | Traditional | 19 (100) | 0 | 0 | 19 |
| | Investigation | 21 (100) | 0 | 0 | 21 |
| 3 | Traditional | 19 (100) | 0 | 0 | 19 |
| | Investigation | 20 (95.2) | 1 (4.8) | 0 | 21 |
| 4 | Traditional | 18 (100) | 0 | 0 | 18 |
| | Investigation | 18 (94.7) | 0 | 1 (5.3) | 19 |
| 5 | Traditional | 16 (94.1) | 1 (5.9) | 0 | 17 |
| | Investigation | 15 (93.8) | 0 | 1 (6.3) | 16 |
| Total | Traditional | 91 (98.9) | 1 (1.1) | 0 | 92 |
| | Investigation | 95 (96.9) | 1 (1.0) | 2 (2.0) | 98 |

**Supplemental Table 6: Elbow Flexion Test Right Arm** [10]

| Visit | Group | Negative | Positive | Missing/Not Tested | Total |
|---|---|---|---|---|---|
| 1 | Traditional | 19 (100) | 0 | 0 | 19 |
| | Investigation | 21 (100) | 0 | 0 | 21 |
| 2 | Traditional | 19 (100) | 0 | 0 | 19 |
| | Investigation | 21 (100) | 0 | 0 | 21 |
| 3 | Traditional | 18 (94.7) | 1 (5.3) | 0 | 19 |
| | Investigation | 21 (100) | 0 | 0 | 21 |
| 4 | Traditional | 18 (100) | 0 | 0 | 18 |
| | Investigation | 18 (94.7) | 0 | 1 (5.3) | 19 |
| 5 | Traditional | 16 (94.1) | 1 (5.9) | 0 | 17 |
| | Investigation | 15 (93.8) | 0 | 1 (6.3) | 16 |
| Total | Traditional | 90 (97.8) | 2 (2.3) | 0 | 92 |
| | Investigation | 96 (98.0) | 0 | 2 (2.0) | 98 |

**Supplemental Table 7: Elbow Flexion Test Left Arm** [10]

| Visit | Group | Negative | Positive | Missing/Not Tested | Total |
|---|---|---|---|---|---|
| 1 | Traditional | 19 (100) | 0 | 0 | 19 |
| | Investigation | 21 (100) | 0 | 0 | 21 |
| 2 | Traditional | 19 (100) | 0 | 0 | 19 |
| | Investigation | 20 (95.2) | 1 (4.8) | 0 | 21 |
| 3 | Traditional | 17 (89.4) | 2 | 0 | 19 |
| | Investigation | 21 (100) | 0 | 0 | 21 |
| 4 | Traditional | 17 (94.4) | 1 (5.6) | 0 | 18 |
| | Investigation | 18 (94.7) | 0 | 1 (5.3) | 19 |
| 5 | Traditional | 16 (94.1) | 1 (5.9) | 0 | 17 |
| | Investigation | 15 (93.8) | 0 | 1 (6.3) | 16 |
| Total | Traditional | 88 (95.7) | 4 (4.3) | 0 | 92 |
| | Investigation | 95 (6.9) | 1 (1.0) | 2 (2.0) | 98 |

**Supplemental Table 8: Tinel Test Right Volar Wrist** [9]

| Visit | Group | Negative | Positive | Missing/Not Tested | Total |
|---|---|---|---|---|---|
| 1 | Traditional | 19 (100) | 0 | 0 | 19 |
| | Investigation | 21 (100) | 0 | 0 | 21 |
| 2 | Traditional | 19 (100) | 0 | 0 | 19 |
| | Investigation | 21 (100) | 0 | 0 | 21 |
| 3 | Traditional | 19 (100) | 0 | 0 | 19 |
| | Investigation | 21 (100) | 0 | 0 | 21 |
| 4 | Traditional | 18 (100) | 0 | 0 | 18 |
| | Investigation | 19 (100) | 0 | 0 | 19 |
| 5 | Traditional | 17 (100) | 0 | 0 | 17 |
| | Investigation | 15 (93.8) | 0 | 1 (6.3) | 16 |
| Total | Traditional | 92 (100) | 0 | 0 | 92 |
| | Investigation | 97 (99.0) | 0 | 1 (1.0) | 98 |

**Supplemental Table 9. Tinel Test Left Volar Wrist** [9]

| Visit | Group | Negative | Positive | Missing/Not Tested | Total |
|---|---|---|---|---|---|
| 1 | Traditional | 19 (100) | 0 | 0 | 19 |
| | Investigation | 20 (95.2) | 1 (4.8) | 0 | 21 |
| 2 | Traditional | 19 (100) | 0 | 0 | 19 |
| | Investigation | 21 (100) | 0 | 0 | 21 |
| 3 | Traditional | 18 (94.7) | 1 (5.3) | 0 | 19 |
| | Investigation | 21 (100) | 0 | 0 | 21 |
| 4 | Traditional | 18 (100) | 0 | 0 | 18 |
| | Investigation | 19 (100) | 0 | 0 | 19 |
| 5 | Traditional | 17 (100) | 0 | 0 | 17 |
| | Investigation | 15 (93.8) | 0 | 1 (6.3) | 16 |
| Total | Traditional | 91 (98.9) | 1 | 0 | 92 |
| | Investigation | 96 (98.0) | 1 (1.0) | 1 (1.0) | 98 |

**Supplemental Table 10: Tinel Test Right Cubital Tunnel** [11]

| Visit | Group | Negative | Positive | Missing/Not Tested | Total |
|---|---|---|---|---|---|
| 1 | Traditional | 19 (100) | 0 | 0 | 19 |
| | Investigation | 21 (100) | 0 | 0 | 21 |
| 2 | Traditional | 19 (100) | 0 | 0 | 19 |
| | Investigation | 21 (100) | 0 | 0 | 21 |
| 3 | Traditional | 18 (100) | 1 (5.3) | 0 | 19 |
| | Investigation | 20 (95.2) | 1 (4.8) | 0 | 21 |
| 4 | Traditional | 17 (94.4) | 1 (5.6) | 0 | 18 |
| | Investigation | 19 (100) | 0 | 0 | 19 |
| 5 | Traditional | 17 (100) | 0 | 0 | 17 |
| | Investigation | 15 (93.8) | 0 | 1 (6.3) | 16 |
| Total | Traditional | 90 (97.8) | 2 (2.3) | 0 | 92 |
| | Investigation | 96 (98.0) | 1 (1.0) | 1 (1.0) | 98 |

**Supplemental Table 11: Tinel Test Left Cubital Tunnel** [11]

| Visit | Group | Negative | Positive | Missing/Not Tested | Total |
|---|---|---|---|---|---|
| 1 | Traditional | 19 (100) | 0 | 0 | 19 |
| | Investigation | 20 (95.2) | 1 (4.8) | 0 | 21 |
| 2 | Traditional | 19 (100) | 0 | 0 | 19 |
| | Investigation | 21 (100) | 0 | 0 | 21 |
| 3 | Traditional | 18 (94.7) | 1 (5.3) | 0 | 19 |
| | Investigation | 21 (100) | 0 | 0 | 21 |
| 4 | Traditional | 17 (94.4) | 1 (5.6) | 0 | 18 |
| | Investigation | 19 (100) | 0 | 0 | 19 |
| 5 | Traditional | 16 (94.1) | 1 (5.9) | 0 | 17 |
| | Investigation | 15 (93.8) | 0 | 1 (6.3) | 16 |
| Total | Traditional | 89 (96.7) | 3 (3.3) | 0 | 92 |
| | Investigation | 96 (98.0) | 1 (1.0) | 1 (1.0) | 98 |

**References**


1. Kennedy CA, Beaton DE, Solway S, McConnell S, Bombardier C. *Disabilities of the Arm, Shoulder and Hand (DASH). The DASH and QuickDASH Outcome Measure User's Manual*. 3 ed. Toronto: Institute for Work & Health; 2011.

2. Beaton DE, Wright JG, Katz JN; Upper Extremity Collaborative Group. Development of the QuickDASH: comparison of three item-reduction approaches. *J Bone Joint Surg Am*. 2005;87(5):1038-1046. doi:10.2106/JBJS.D.02060

3. Rayan GM. Thoracic outlet syndrome. *J Shoulder Elbow Surg*. 1998;7(4):440-451. doi:10.1016/s1058-2746(98)90042-8

4. Pratt NE. Neurovascular entrapment in the regions of the shoulder and posterior triangle of the neck. *Phys Ther*. 1986;66(12):1894-1900. doi:10.1093/ptj/66.12.1894

5. Brantigan CO, Roos DB. Diagnosing thoracic outlet syndrome. *Hand Clin*. 2004;20(1):27-36. doi:10.1016/s0749-0712(03)00080-5

6. Oates SD, Daley RA. Thoracic outlet syndrome. *Hand Clin*. 1996;12(4):705-718.

7. Phalen GS. The carpal-tunnel syndrome. Seventeen years' experience in diagnosis and treatment of six hundred fifty-four hands. *J Bone Joint Surg Am*. 1966;48(2):211-228.

8. MacDermid JC, Doherty T. Clinical and electrodiagnostic testing of carpal tunnel syndrome: a narrative review. *J Orthop Sports Phys Ther*. 2004;34(10):565-588. doi:10.2519/jospt.2004.34.10.565

9. Evans RB. Therapist's Management of Carpal Tunnel Syndrome: A Practical Approach. In: Skirven TM, Osterman AL, Fedorczyk JM, Amadio PC, eds. *Rehabilitation of the Hand and Upper Extremity*. 6 ed. Philadelphia: Mosby; 2011. 666-677.

10. Novak CB, Mackinnon SE. Evaluation of nerve injury and nerve compression in the upper quadrant. *J Hand Ther*. 2005;18(2):230-240. doi:10.1197/j.jht.2005.02.002

11. Rekant MS. Diagnosis and Surgical Management of Cubital Tunnel Syndrome. In: Skirven TM, Osterman AL, Fedorczyk JM, Amadio PC, eds. *Rehabilitation of the Hand and Upper Extremity*. 6 ed. Philadelphia: Mosby; 2012. 678-685.

12. Bell-Krotoski J, Tomancik E. The repeatability of testing with Semmes-Weinstein monofilaments. *J Hand Surg Am*. 1987;12(1):155-161. doi:10.1016/s0363-5023(87)80189-2

13. Bell-Krotoski JA, Buford WL Jr. The force/time relationship of clinically used sensory testing instruments. *J Hand Ther*. 1997;10(4):297-309. doi:10.1016/s0894-1130(97)80045-2

14. Bell-Krotoski JA. Sensibility Testing History, Instrumentation, and Clinical Procedures. In: Skirven TM, Osterman AL, Fedorczyk JM, Amadio PC, eds. *Rehabilitation of the Hand and Upper Extremity*. 6 ed. Philadelphia: Mosby; 2012. 132-151.

15. Strauch B, Lang A, Ferder M, Keyes-Ford M, Freeman K, Newstein D. The ten test. *Plast Reconstr Surg*. 1997;99(4):1074-1078. doi:10.1097/00006534-199704000-00023

16. Dellon AL. The moving two-point discrimination test: clinical evaluation of the quickly adapting fiber/receptor system. *J Hand Surg Am*. 1978;3(5):474-481. doi:10.1016/s0363-5023(78)80143-9

17. Moberg E. Criticism and study of methods for examining sensibility in the hand. *Neurology*. 1962;12:8-19. doi:10.1212/wnl.12.1.8

18. Mackinnon SE, Dellon AL. Two-point discrimination tester. *J Hand Surg Am*. 1985;10(6 Pt 1):906-907. doi:10.1016/s0363-5023(85)80173-8

19. Bell-Krotoski JA. Sensibility Testing: Current Concepts. In: Hunter JM, Mackin EJ, Callahan AD, eds. *Rehabilitation of the Hand: Surgery and Therapy*. 4 ed. St. Louis: Mosby; 1995. 109-128.

20. Bell-Krotoski JA. Sensibility Testing. In: Burke SL, Higgins JP, McClinton MA, Saunders RJ, Valdata L, eds. *Hand and Upper Extremity Rehabilitation*. 3 ed. St. Louis: Churchill Livingstonw; 2005. 53-85.

21. Sriwatanakul K, Kelvie W, Lasagna L, Calimlim JF, Weis OF, Mehta G. Studies with different types of visual analog scales for measurement of pain. *Clin Pharmacol Ther*. 1983;34(2):234-239. doi:10.1038/clpt.1983.159

22. Bijur PE, Silver W, Gallagher EJ. Reliability of the visual analog scale for measurement of acute pain. *Acad Emerg Med*. 2001;8(12):1153-1157. doi:10.1111/j.1553-2712.2001.tb01132.x
